# Retinal and Optic Disc Vascular Changes in Patients Using Long-Term Tadalafil: A Prospective Non-Randomized Matched-Pair Study

**DOI:** 10.3390/diagnostics11050802

**Published:** 2021-04-28

**Authors:** Marco Capece, Daniela Montorio, Chiara Comune, Achille Aveta, Alberto Melchionna, Giuseppe Celentano, Ciro Imbimbo, Felice Crocetto, Gianluigi Califano, Gilda Cennamo

**Affiliations:** 1 Department of Neurosciences, Reproductive Sciences and Dentistry, University of Naples “Federico II”, 80131 Naples, Italy; drmarcocapece@gmail.com (M.C.); da.montorio@gmail.com (D.M.); chiara.comune.cc@gmail.com (C.C.); achille-aveta@hotmail.it (A.A.); alb.melchionna@gmail.com (A.M.); dr.giuseppecelentano@gmail.com (G.C.); ciro.imbimbo@unina.it (C.I.); felice.crocetto@gmail.com (F.C.); gianl.califano2@gmail.com (G.C.); 2 Eye Clinic, Public Health Department, University of Naples “Federico II”, 80131 Naples, Italy

**Keywords:** tadalafil, erectile dysfunction, radical prostatectomy, retinal vascularity, eye

## Abstract

Retinal, choroidal and optic disc vascularity has never been evaluated in patients taking PDE5is long-term. The aim of our study was to evaluate the neurostructural and vascular changes after long-term use of tadalafil, using spectral domain (SD)-optical coherence tomography (OCT) and optical coherence tomography angiography (OCTA). In the present clinical trial, 27 patients who have been taking tadalafil 20 mg on alternate days (OAD) for at least 6 months (Group A) were enrolled. The matched group consisted of 27 healthy men (Group B). Both groups of patients underwent SD-OCT to study ganglion cell complex (GCC), retinal nerve fiber layer (RNFL) and choroidal thickness and OCTA for the evaluation of superficial capillary plexus (SCP), deep capillary plexus (DCP), choriocapillaris (CC) and radial peripapillary capillary (RPC). A reduction in SCP, DCP and RPC vessel density was found in patients using tadalafil long-term. Retinal and optic disc toxicity may be detected using modifications of capillary vessel density. Further studies are needed to investigate the possibility of a causal association.

## 1. Introduction

Phosphodiesterase type 5 inhibitors (PDE5is) are the first-line treatment for erectile dysfunction (ED) [1,2]. Despite PDE5is mainly blocking phosphodiesterase 5 (PDE5), a minimal affinity to other types of PDEs has been previously confirmed [2]. It has been established that PDE5is also suspend PDE2-4, PDE7-11 and PDE6 activities (10-fold less potent than on PDE5) [3]. The latter control the level of cyclic guanosine monophosphate (cGMP) in retinal rod and cone cells for visual signal transduction. Indeed the activation of PDE6 leads to the reduction in cGMP levels in photoreceptors, which in turn determines the closure of sodium channels in the outer segment cell membrane, the membrane hyperpolarization and the inception of light transduction [3,4,5]. Thus visual symptoms are well-known side effects of PDE5is; nevertheless, they do not seem to cause permanent toxicity on chorioretinal tissues and photoreceptors [3].

Nonarteritic anterior ischemic optic neuropathy (NAION) has been described in men using PDE5is in many studies [6,7]. NAION is a rare severe ocular disease, most likely induced by hypoperfusion and consequent ischemia of the optic nerve [8]. Tadalafil, a unique long half-life PDE5i, has been used for years to treat erectile dysfunction, benign prostatic hyperplasia and pulmonary hypertension [9,10,11]. However, its administration has been sometimes associated with ocular side effects. In fact, it has been implicated in the development of thickened and hyperreflective areas in the photoreceptors of the superficial and deep segments of the retina, simulating a serous retinal detachment-like appearance [12].

No studies have been designed to evaluate the retinal and choroidal vascularity in patients taking PDE5is long-term. Human retina consists of 10 layers supplied by two vascular beds: three retinal capillary plexuses and choroid. Retinal capillaries supply the inner five layers and accommodate visual function, whereas the outer five layers of the retina are almost avascular and receive oxygen and nutrients from the choroidal vessels. Evaluating and quantifying vascular density or blood flow at different retinal layers in patients undergoing long-term use of PDE5is may be useful to assess the integrity of such structures [4,5].

Historically the evaluation of eye vascularity has always been performed using retinal angiography [13]. However, considering the possible side effects of fluorescein and the inconvenience of the procedure, it has been recently outweighed by a new, non-invasive imaging technique: the optical coherence tomography angiography (OCTA) [13]. This novel technique provides depth-resolved images of retinal and choroidal vessels, without intravenous dye agent injection, unlike more traditional retinal fluorescein and green indocyanine angiographies. OCTA is safe, repeatable and allows the separate visualization of the superficial and deep retinal capillary plexuses and the choriocapillaris [14].

The aim of our study was to evaluate the neurostructural and vascular features of the retina and choroid, using spectral domain (SD)-optical coherence tomography (OCT) and OCTA, respectively, in patients undergoing long-term therapy with tadalafil.

## 2. Materials and Methods

### 2.1. Study Design

The present study was a prospective, non-randomized, matched-pair clinical trial of a cohort of 27 patients who were treated with tadalafil 20 mg on alternate days (OAD) after nerve-sparing robotic radical prostatectomy (NS-RARP) for prostate cancer (Group A). Patients were enrolled at the PCaFu outpatient clinic (Prostate Cancer Follow-up) of the University of Naples “Federico II” from November 2019 to February 2020. Inclusion criteria were:

(a) Patients suffering from iatrogenic ED < 70 years old;
(b) Continuous administration of tadalafil 20 mg OAD for >6 months;
(c) No evidence of retinopathy.

Each patient matching the criteria underwent an andrological and ocular assessment including: hormonal evaluation, erectile function evaluation, IIEF-5 questionnaire, the measurement of best-corrected visual acuity (BCVA) based on the Early Treatment Diabetic Retinopathy Study (ETDRS) charts, slit-lamp and fundus examination, evaluation of the structural SD-OCT parameters (ganglion cell complex and retinal nerve fiber layer, subfoveal choroidal thickness) and optical coherence tomography angiography (OCTA).

Exclusion criteria included previous treatment for erectile dysfunction before surgery, hypertension, heart diseases, diabetes, drug intake for these systemic pathologies, previous ocular surgery, congenital eye disease, myopia greater than 6 diopters, retinal vascular diseases, macular diseases, history of glaucoma and significant lens opacities to avoid low-quality OCT and OCTA images.

Each analyzed patient included in the study was matched with a control, naïve for tadalafil or any other PDE5is, applying the following matching criteria: age, comorbidities such as dyslipidemia (<220 mg/dL) and smoking habits (≤10 cigarettes per day). The control group constituted 54 eyes of 27 healthy subjects that presented a normal ophthalmological evaluation without any history of intraocular surgery or retinal pathologic features (Group B).

Both groups of patients underwent single-time SD-OCT and OCTA measurements, performed by two masked examiners (CC, DM) and a senior expert (GC).

The study adhered to the tenets of the Declaration of Helsinki. Written informed consent was obtained from the patients enrolled in the study. The research protocol was registered on Clinical Trials.gov (25 July 2020) (NCT04491773).

### 2.2. Study Techniques

#### 2.2.1. Spectral Domain-Optical Coherence Tomography

SD-OCT (software RTVue XR version 2017.1.0.151, Optovue Inc., Fremont, CA, USA) examined the retinal nerve fiber layer (RNFL) and ganglion cell complex (GCC) thickness in all patients. The optic nerve head (ONH) analysis measured the RNFL thickness, calculated along a 3.45-mm diameter circle around the optic disc. The GCC thickness was measured from the internal limiting membrane to the outer boundary of the inner plexiform layer in a 7 × 7 mm^2^ grid of the macula centered 1-mm temporal to the fovea [15]. OCTA images with a signal strength index (SSI) less than 80 and residual motion artifacts were excluded from the analysis.

#### 2.2.2. Subfoveal Choroidal Thickness Measurement

The subfoveal choroidal thickness (SFCT) was analyzed using SD-OCT (software RTVue XR version 2017.1.0.151, Optovue Inc., Fremont, CA, USA). A manual linear measurement evaluated the subfoveal region between the outer border of Bruch’s membrane and the most posterior identifiable aspect of the choroidal–scleral interface. The analysis excluded the scans with quality score of less than 20 [16].

#### 2.2.3. Optical Coherence Tomography Angiography

All the subjects underwent OCTA (Optovue AngioVue System, software ReVue XR version 2017.1.0.151, Optovue Inc., Fremont, CA, USA).

Macular capillary network was evaluated in scans centered on the fovea by performing a 6 mm × 6 mm area divided, according to the ETDRS classification of diabetic retinopathy, in whole image, fovea and parafovea.

The AngioAnalyticTM software automatically analyzed the vessel density (VD) in two different retinal vascular networks: superficial capillary plexus (SCP), deep capillary plexus (DCP) and in the choriocapillaris (CC), as previously described [17].

The VD was defined as the percentage area occupied by the microvasculature in whole scan area and in all sections [18].

The VD of the radial peripapillary capillary plexus (RPC), analyzing the whole papillary region, inside the disc and peripapillary region with an area scan of 4.5 *×* 4.5 mm^2^, was automatically calculated by the AngioVue disc mode [19].

The 3D projection artifact removal (PAR) algorithm, included in the software, improved the quality of OCTA images.

OCTA images with an SSI less than 80 and residual motion artifacts were excluded from the analysis.

### 2.3. Statistical Analysis

The Statistical Package for Social Sciences (Version 20.0 for Windows; SPSS Inc., Chicago, IL, USA) was used for statistical analysis. The sample size (at least 27 eyes for each group) was determined from the results of our preliminary data to detect, with an alpha of 0.05 and 80% power, a 3% difference in the rate of changes in retinal vessel density at OCTA. The Shapiro–Wilk test was performed to test for normality, and all variables were not normally distributed due to insufficient data. The differences in OCTA and SD-OCT parameters between the patients and the healthy controls were evaluated by Mann–Whitney test. A *p* value of <0.05 was considered statistically significant.

## 3. Results

In the present study, 27 patients taking tadalafil 20 mg OAD were enrolled in Group A and matched with 27 healthy patients in Group B, for a total of fifty-four patients (108 eyes) that were examined and compared. The mean time of tadalafil administration was 11 ± 5 months (range 6–24 months).

Mean age of Group A was 63.7 ± 6.9 years, whereas in Group B, it was 64.1 ± 9 years. There were no significant differences between the two groups for the matching criteria (dyslipidemia *p* = 0.761; smoking habits *p* = 0.483, testosterone levels *p* = 0.644) for age (*p* = 0.933) and BCVA (*p* = 0.301) between the two groups. A statistically significant difference was found in the IIEF-5 questionnaire (6.52 ± 1.25 vs. 22.0 ± 1.7 in Groups A and B, respectively, *p* < 0.001). The demographic and clinical characteristics of both groups are reported in Table 1.

In SD-OCT exam, there were no statistically significant differences between Group A and Group B in GCC (average: 98.18 ± 6.27 µm vs. 96.68 ± 8.16 µm; *p* > 0.05) and RNFL parameters (average: 99.94 ± 8.21 µm vs. 98.33 ± 7.89 µm; *p* > 0.05) while the SFCT turned to be significantly thicker in Group A than in the control group (*p* < 0.001) (Table 2).

Regarding the OCTA parameters, the VD of the SCP was significantly lower in Group A with respect to Group B in whole image (51.63 ± 4.75% vs. 48.54 ± 4.22%; *p* = 0.002), parafovea area (53.63 ± 2.18% vs. 50.77 ± 6.97%; *p* = 0.013) and fovea area (26.19 ± 4.58% vs. 22.53 ± 8.17%; *p* = 0.012).

Likewise, Group A showed a statistically significant reduction in VD of the DCP compared to Group B in whole image (54.20 ± 2.82% vs. 51.20 ± 7.25%; *p* = 0.028), parafoveal area (57.14 ± 2.52% vs. 54.31 ± 6.74%; *p* = 0.019) and foveal area (44.28 ± 4.06% vs. 40.29 ± 9.61%; *p* = 0.02).

The VD of the RPC resulted statistically reduced in Group A compared to Group B in whole image (50.87 ± 6.62% vs. 47.95 ± 2.53%; *p* = 0.044), inside disc (53.52 ± 3.33% vs. 51.32 ± 4.97%; *p* = 0.022) and peripapillary region (52.82 ± 4.21% vs. 50.41 ± 2.64%; *p* = 0.001).

**Figure 1:**
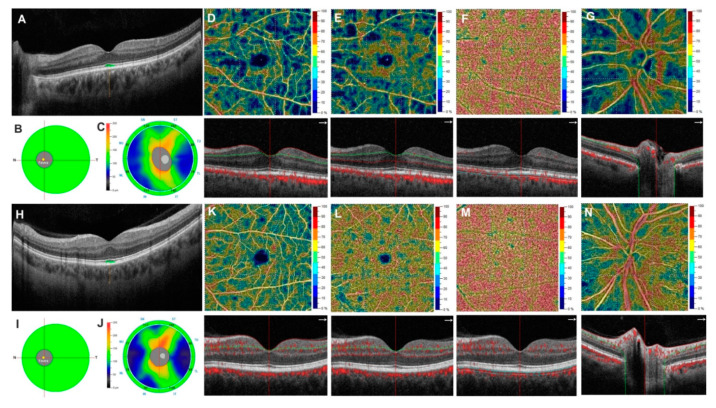
Top row. Left eye of a 65-year-old patient undergoing chronic long-term therapy with tadalafil shows an increased subfoveal choroidal thickness (SFCT) (**A**) and a normal ganglion cell complex (GCC) and retinal nerve fiber layer (RNFL) parameters (**B**,**C**). In OCTA images, the retinal superficial, deep capillary plexuses (**D**,**E**) and the radial peripapillary capillary plexus (**G**) reveal areas of reduced vessel density and no change in choriocapillaris vessel density (**F**). Bottom Row. Left eye of a 63-year-old healthy subject (part of the control group) presents normal SFCT (**H**) and normal GCC and RNFL parameters (**I**,**J**). OCTA images reveal normal vessel density in retinal superficial, deep capillary plexuses, choriocapillaris and radial peripapillary capillary plexus (**K**–**N**).

On the other hand, there were no statistically significant differences in the VD of CC between Group A and Group B (whole image: 73.12 ± 3.19% vs. 73.16 ± 3.14%; *p* = 0.975; parafovea region: 71.62 ± 5.16% vs. 71.47 ± 3.57%; *p* = 0.393; fovea region: 70.03 ± 4.29% vs. 69.87 ± 5.60%; *p* = 0.601) (Table 3 and Figure 1).

## 4. Discussion

PDE5is are used to enhance the effect of nitric oxide in increasing the blood flow into the corpus cavernosum. These agents only affect the response to sexual stimulation but do not directly cause penile erections. In 2003, tadalafil was approved by the Food and Drug Administration for the treatment of erectile dysfunction. The most known adverse effects are headache, flushing, dyspepsia and nasal congestion, whereas ocular side effects are less frequent. Among these, short-term impaired color vision [5,12], blurred vision [7] and increased brightness [20] have been reported. In a few cases, the use of tadalafil led to ischemic optic neuropathy [7] and retinal artery and vein occlusion too [20]. To the best of our knowledge, this is the first study on retinal and optic disc vascular density in patients treated with tadalafil using OCTA.

The association between use of tadalafil and ocular complications is not well known. Previous studies evaluated the effects of sildenafil citrate on choroidal thickness and vascular flow because of the anatomical similarities between corpus cavernosum and choroid [21,22]. Such trials confirmed that sildenafil can increase the blood flow of the short posterior ciliary arteries and the choroidal thickness [21,22,23,24]. Yiu et al. found that choroidal thickness increased 1 and 3 h after a single dose of sildenafil 100 mg in patients affected by age-related macular degeneration (AMD) [23]. Such increment was also seen in age-matched controls, although smaller than in studies on younger patients [23,24,25]. The choroidal thickening was attributed to the expansion of the stroma rather than vascular dilatation [23]. Recently Aslan et al. observed an increase in small-choroidal-vessel-layer (choriocapillaris plus Sattler’s layer) and total choroidal thickness after 3 months of daily oral 5 mg tadalafil in patients with or without erectile dysfunction [26].

A histological study on the chronic effect of tadalafil on the retina in rats demonstrated dilated blood capillaries in the inner retinal layers [27].

To better analyze the eye vascularity of patients taking tadalafil, we suggested the use of OCTA to analyze the vessel density of retinal capillary plexuses (SCP, DCP and RPC) and CC. We compared patients using tadalafil for more than 6 months and healthy controls. In the present study, patients enrolled started erectile function rehabilitation therapy after radical prostatectomy according to a regional protocol that consists of tadalafil 20 mg every other day. Such protocol leads to the intake of 70 mg of tadalafil per week, which is twice the maximum amount recommended by guidelines [28]. Our results highlight that patients undergoing long-term tadalafil treatment show a significantly lower microvascular VD in both SCP and DCP compared to the control group. On the contrary, no differences in CC vascular density were demonstrated between the two groups. In accord with previous studies, we found an increase in choroidal thickness in patients taking tadalafil. However, considering the lack of baseline evaluation we cannot hypothesize a causal effect between the use of PDE5is and the vascular impairment.

Our data reflect possible damage of the retinal capillaries; notwithstanding, the choriocapillaris remains intact. Such discrepancy in our results may be related to the different ultrastructure of the CC that presents fenestrations [29]. In addition to that, no studies have confirmed the presence of PDE in such a layer, implicating the unaltered CC structure. These findings are in contrast with a previous hypothesis regarding the possible effects of PDE5 being only on choroidal circulation [21,22,24]. In fact, retinal vascularization has never been studied in previous studies.

Moreover, we found that VD of RCP is dramatically reduced in the group of patients taking tadalafil. These results on optic disc VD may be correlated with the reported association of non-arteritic anterior ischemic optic neuropathy (NAION) and PDE5is [29,30,31]. Campbell et al. found an approximately twofold increased risk of acute NAION in men using PDE5is compared to naïve patients [6].

Unlike NAION, we did not observe alterations in RNFL and GCC thickness in Group A. However, such structural changes could be secondary to capillary damage, and thus the decreased VD of RCP could be considered an early precursor of NAION or other glaucoma-like neuropathies. Indeed, previous studies have reported a correlation between the use of PDE5is and glaucoma hypothesizing a possible role in increased intraocular pressure [32]. However, none of these papers have taken into consideration the possibility of a progressive degeneration of the whole neurovascular ultrastructure due to the reduction in RCP vessel density.

The present study has a limitation that impedes possible speculations. The lack of baseline ocular parameters prevents the possibility of determining and quantifying the possible decline and the realization of degeneration curves, creating some black holes that should be further investigated. Furthermore, the relatively small number of patients and controls represents another potential limitation of this study. Moreover, another potential limitation could be related to the matching criteria of the control group. Indeed, patients included in this group are matched for most of the parameters but not for prostate cancer disease.

Further studies are necessary in order to determine whether RNFL and GCC will be damaged after a longer administration of tadalafil. Moreover, we cannot determine whether these findings are directly caused by long-term tadalafil administration or merely a selection population bias. In addition to that, it would be interesting to evaluate the relationship between the retinal structural and vascular parameters in these patients.

The lack of a baseline evaluation does not allow to confirm a causal effect between PDE5i use and vascular compromise. It may be useful, in the future, to evaluate patients at baseline with OCTA before NS-RARP and use subjects operated on but not treated with tadalafil as a control group.

In the end, new clinical and preclinical trials should be designed in order to determine the effects of long-term tadalafil administration (or possible transitory nature of the effects). OCT and OCTA of macular and optic disc seem to be excellent non-invasive diagnostic tools to evaluate the eye vascularity in such patients.

## Figures and Tables

**Table 1:** Demographic and clinical characteristics in controls and patients.

	Group B	Group A	*p* Value
Eye (n.)	54	54	-
Age (years)	64.1 ± 9	63.7 ± 6.9	0.933
BCVA (logMAR)	0.06 ± 0.08	0.04 ± 0.08	0.301
Mean spherical equivalent (diopters)	−0.50 ± 1.75	0.75 ± 1.30	0.532
Dyslipidemia (n. subjects)	7	8	0.761
Smoking habits (n. subjects)	4	6	0.483

Data expressed as mean ± SD. BCVA: best corrected visual acuity; logMAR: logarithm of the minimum angle of resolution.

**Table 2:** Differences in OCT parameters between controls and patients.

	Group B	Group A	*p* Value
GCC (µm)			
Average	98.18 ± 6.27	96.68 ± 8.16	0.449
Superior	96.90 ± 6.15	96.22 ± 8.23	0.897
Inferior	98.05 ± 6.06	97.11 ± 8.25	0.680
RNFL (µm)			
Average	99.94 ± 8.21	98.33 ± 7.89	0.625
Superior	102.74 ± 8.63	101.16 ± 8.87	0.518
Inferior	96.40 ± 6.80	95.33 ± 8.41	0.493
Subfoveal choroidal thickness (µm)	255.38 ± 64.24	389.05 ± 76.67	<0.001

Data expressed as mean ± SD. GCC: ganglion cell complex; RNFL: retinal nerve fiber layer. The Mann–Whitney U test, *p* < 0.05.

**Table 3:** Differences in OCT angiography vessel density between controls and patients.

	Group B	Group A	*p* Value
SPC (%)			
Whole image	51.63 ± 4.75	48.54 ± 4.22	0.002
Parafovea	53.63 ± 2.18	50.77 ± 6.97	0.013
Fovea	26.19 ± 4.58	22.53 ± 8.17	0.012
DPC (%)			
Whole image	54.20 ± 2.82	51.20 ± 7.25	0.028
Parafovea	57.14 ± 2.52	54.31 ± 6.74	0.019
Fovea	44.28 ± 4.06	40.29 ± 9.61	0.021
CC (%)			
Whole image	73.12 ± 3.19	73.16 ± 3.14	0.975
Parafovea	71.62 ± 5.16	71.47 ± 3.57	0.393
Fovea	70.03 ± 4.29	69.87 ± 5.60	0.601
RPC (%)			
Whole image	50.87 ± 6.62	47.95 ± 2.53	0.044
Inside Disc	53.52 ± 3.33	51.32 ± 4.97	0.022
Peripapillary	52.82 ± 4.21	50.41 ± 2.64	0.001

Data are expressed as mean ± SD. SCP: superficial capillary plexus, DCP: deep capillary plexus, CC: choriocapillaris, RPC: radial peripapillary capillary. The Mann–Whitney U test, *p* < 0.05.

## Data Availability

The data presented in this study are available on request from the corresponding author. The data are not publicly available due to patients’ privacy.
